# Early pancreatic islet fate and maturation is controlled through RBP-Jκ

**DOI:** 10.1038/srep26874

**Published:** 2016-05-31

**Authors:** Corentin Cras-Méneur, Megan Conlon, Yaqing Zhang, Marina Pasca Di Magliano, Ernesto Bernal-Mizrachi

**Affiliations:** 1 University of Michigan in Ann Arbor, Internal Medicine Department, MEND Division Brehm Tower, 1000 Wall St, Ann Arbor, MI 48105-1912, USA; 2 University of Michigan in Ann Arbor, Department of Surgery, General Surgery Division 4304 Cancer Center, 1500 E. Medical Center Drive, Ann Arbor MI 48109-5936, USA; 3 University of Miami Miller School of Medicine, Department of General Internal Medicine, Division of Endocrinology, Diabetes and Metabolism 1400 NW 10th Ave, Miami, FL 33136-1031, USA.

## Abstract

Notch signaling is known to control early pancreatic differentiation through Ngn3 repression. In later stages, downstream of Notch, the Presenilins are still required to maintain the endocrine fate allocation. Amongst their multiple targets, it remains unclear which one actually controls the maintenance of the fate of the early islets. Conditional deletions of the Notch effector RBP-Jκ with lineage tracing in Presenilin-deficient endocrine progenitors, demonstrated that this factor is central to the control of the fate through a non-canonical Notch mechanism. RBP-Jκ mice exhibit normal islet morphogenesis and function, however, a fraction of the progenitors fails to differentiate and develop into disorganized masses resembling acinar to ductal metaplasia and chronic pancreatitis. A subsequent deletion of RBP-Jκ in forming β-cells led to the transdifferentiation into the other endocrine cells types, indicating that this factor still mediates the maintenance of the fate within the endocrine lineage itself. These results highlight the dual importance of Notch signaling for the endocrine lineage. Even after Ngn3 expression, Notch activity is required to maintain both fate and maturation of the Ngn3 progenitors. In a subset of the cells, these alterations of Notch signaling halt their differentiation and leads to acinar to ductal metaplasia.

The pancreas develops from Pdx1 and Ptf1a-expressing progenitors in the foregut. An integrated network of transcription factors acts in concert during pancreatogenesis to carefully balance the maturation of these progenitors between the endocrine, acinar, and ductal lineage. The molecular steps involved in their maturation into Insulin-producing islets have been the subjects of many studies. Notch signaling has been described as a master regulator of the initial endocrine to acinar decision, regulating the expression of the endocrine precursor marker Ngn3 (reviewed in)^1^.

The Notch genes encode conserved, membrane-bound receptors mediating short-range cell-cell communications. Notch proteins are proteolytically processed in response to ligand binding reviewed in^2^. Intramembrane proteolysis and activation of all Notch receptors is mediated by the Presenilins, at the core of the γ-Secretase complex. Once cleaved, the NICD enters the nucleus where it displaces co-repressors, recruits Mastermind and RBP-Jκ, thereby converting a transcriptional repressor, into an activator complex^3^. During pancreatic organogenesis, Notch signaling antagonizes Ngn3 expression, while allowing the Ptf1a/p48 complex to direct the early progenitors towards acinar differentiation^4^,^5^,^6^. Even though the involvement of Notch signaling in early pancreatogenesis has been established, its potential implication in controlling the maturation of endocrine progenitors after Ngn3 expression remains unclear.

In a previous publication, we inactivated all Notch signaling in endocrine progenitors by deleting both Presenilins using a *Ngn3-Cre*^7^. Unexpectedly, we discovered that Ngn3 progenitors, originally thought to be committed to the endocrine fate, selected the acinar fate when presenilin dose was reduced. Right after Ngn3 is expressed, the γ-Secretase complex promotes, rather than inhibits, the endocrine lineage. The phenotype associated with presenilin-nulls can be attributed in most cases to loss of Notch signaling^8^,^9^,^10^, however, Presenilins have been implicated in multiple additional processes (ErbB4 proteolysis^11^, transport vesicles^12^,^13^,^14^, scaffolds for Akt activation^15^, β-Catenin degradation^16^, Erk activation^17^,^18^…) which have all been previously shown to be involved in pancreatic development^19^,^20^,^21^,^22^,^23^,^24^.

Based on the fact that the Presenilins control of endocrine fate correlates with the dosage of either Presenilins or Notch^7^, this regulation appears to be the result of a competition for a downstream effector rather than a canonical Notch response. Notch and Ptf1a are known to compete to form a complex with RBP-Jκ which defines the late acinar differentiation^4^,^25^. The sustained activation of the Ptf1a/RBP-Jκ complex promotes acinar differentiation^26^,^27^ whereas its dissociation leads to a quick disappearance of Ptf1a expression^28^. While the Ptf1a-RBP-Jκ complex is central to the promotion of the acinar program, a previous publication^29^ has illustrated how a competition between NICD and Ptf1a can controls acinar differentiation in a non-canonical Notch-dependent manner. Our previous publication had demonstrated that the Presenilins still controlled the fate of the endocrine progenitors, even after Ngn3 had been expressed during pancreatic development. Simply lowering Notch signaling in these cells could divert them towards the acinar fate. This non-canonical mechanism, associated to the fact that Ptf1a was still present in these cells strongly suggested that Notch needs to be activated to sequester RBP-Jκ away from Ptf1a in Ngn3 progenitors to prevent the promotion of the acinar program^7^.

In these studies, we demonstrated that indeed RBP-Jκ, the downstream Notch effector, is central to the early fate-maintenance of the endocrine progenitors. Conditional deletion of RBP-Jκ can revert the Presenilin phenotype and maintain the Ngn3 progenitors within their original endocrine lineage again even though all Notch signaling is prevented.

When RBP-Jκ alone is eliminated, even though Notch signaling is then completely blocked, the Ngn3 cells are no longer redirected towards the acinar fate. Furthermore, the majority of the Ngn3 progenitors properly differentiate into functional islets, although a fraction of these cells fail to properly differentiate and persist in adulthood, forming large disorganized masses with features that resemble acinar to ductal metaplasia with premalignant lesions, leading to chronic pancreatitis. Deleting RBP-Jκ in early β-cells also illustrates the importance of this factor in the maintenance of the endocrine fate: in the absence of RBP-Jκ, lineage-tracing reveals that some of these early β-cells are redirected towards the other endocrine lineages within the islets.

These results illustrate that non-canonical Notch signaling is required, not only to prevent a redirection of the cells towards the acinar lineage, but also that RBP-Jκ is later needed for the activation and maintenance of a proper endocrine program.

## Results

### The control of the endocrine fate by the Presenilins is RBP-Jκ dependent

We had previously hypothesized that fate control could result from a competition between Notch Intra Cellular Domain (NICD) and Ptf1a for RBP-Jκ^7^. NICD would be required to sequester RBP-Jκ away from Ptf1a in order to prevent the promotion of the acinar fate in this lineage. To test this original hypothesis, RBP-Jκ was deleted from Presenilin-null Ngn3 cells in mice using lineage tracing (*Ngn3-Cre; Ps1*^*f*/*f*^*; Ps2*^−/−^*; RBP-Jκ*^*f*/*f*^*; Z*/*EG*). In this model, upon expression of the Cre recombinase, the Ngn3 progenitors start constitutively expressing the Enhanced Green Fluorescent Protein (EGFP) fluorescent tag from the LacZ/EGFP (*Z*/*EG*) reporter construct^30^. The reporter allows the tracing of the fate of the Control or Presenilins and RBP-Jκ triple deleted Ngn3 progenitors over time through the expression of the reporter itself. Only cytoplasmic expression of the reporter should be taken into account as the fixation conditions occasionally lead to a non-specific diffusion and nuclear localization of the EGFP as previously reported^31^,^32^.

**Figure 1:**
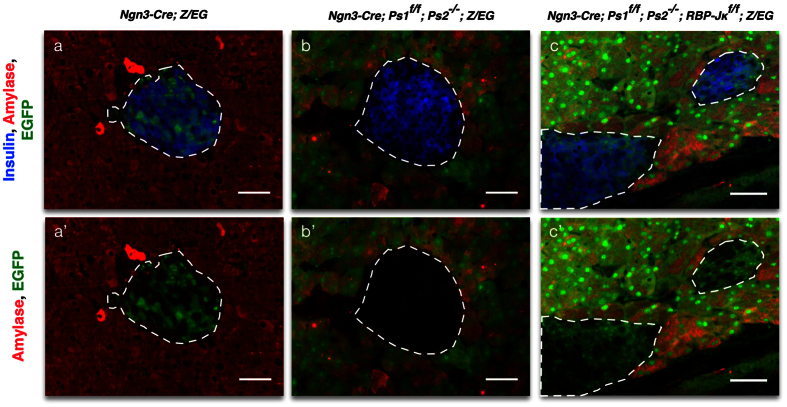
Deletion of RBP-Jκ in the Presenilin mice reverts the redirection of the endocrine fate. Immunostaining for Insulin (blue), Amylase (red) and EGFP (green) in the control (**a**-**a’**), Presenilin (**b**-**b’**) and Presenilin-RBP-Jκ (**c**-**c’**) adult mice. Dotted white lines delimit the islets. Scale bars = 50 μm.

As expected, in the control *Ngn3-Cre; Z*/*EG* adult mice, the EGFP reporter was exclusively found in the islets (Fig. 1a). When both Presenilins are deleted from Ngn3 progenitors (*Ngn3-Cre; Ps1*^*f*/*f*^*; Ps2*^−/−^*; Z*/*EG* mice), EGFP expression is exclusively found in the acinar compartment suggesting that endocrine progenitors are redirected towards the acinar fate as previously described^7^ (Fig. 1b). Elimination of RBP-Jκ from Presenilins null Ngn3 progenitors (*Ngn3-Cre; Ps1*^*f*/*f*^*; Ps2*^−/−^*; RBP-Jκ*^*f*/*f*^*; Z*/*EG*) reverses the phenotype and EGFP expression is, again, observed in islets (Fig. 1c). These animals display some degree of variability in the location of the expression of the fluorescent reporter, especially in the acinar compartment, most likely due to mosaicism and timing of the multiple alleles involved in this model. These studies showed that the redirection of Ngn3 progenitors towards the acinar fate induced by complete inactivation of the Notch signaling pathway was prevented by concomitant deletion of RBP-Jκ.

### Loss of RBP-Jκ in the islets is dispensable for glucose homeostasis

**Figure 2:**
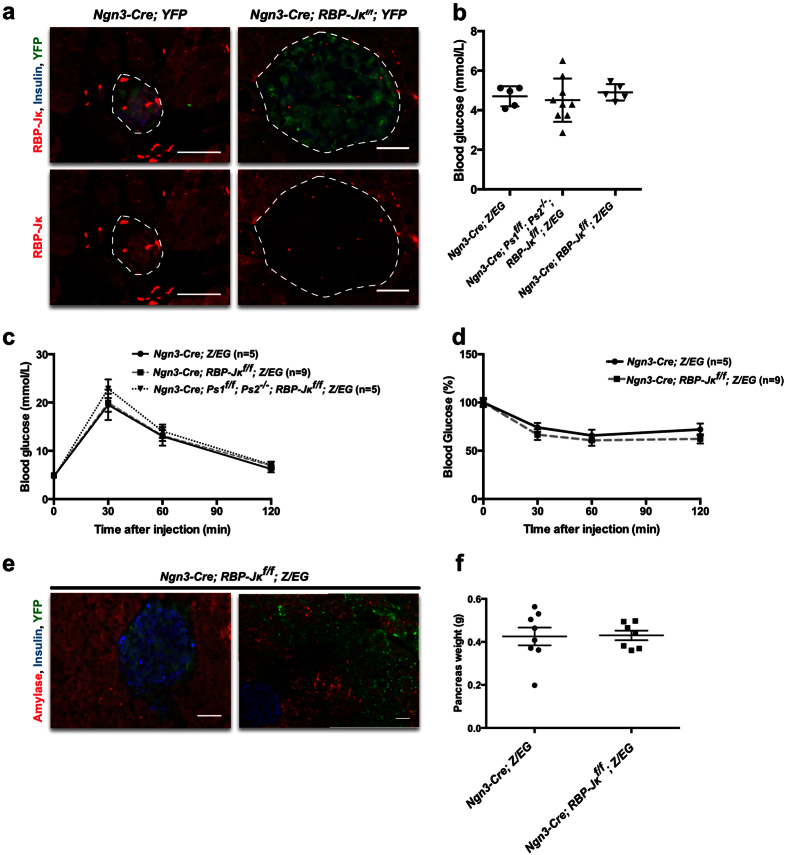
Characterization of the conditional elimination of RBP-Jκ in the Ngn3 progenitors. (**a**) Immunostaining for RBP-Jκ (red), Insulin (blue) and YFP (green) in control and RBP-Jκ adult mice. The lower panel shows immunostaining for RBP-Jκ alone. (**b**) Fasting blood glucoses in 3 months-old control, Presenilin and RBP-Jκ male mice. (**c**) IPGTT in control, Presenilin and RBP-Jκ male mice. (**d**) ITT in control, Presenilin and RBP-Jκ male mice as a percentage compared to t0. (**e**) Immunostaining for Amylase (red), Insulin (blue) and EGFP (green) in the RBP-Jκ mice. (**f**) Pancreas weights for the control and RBP-Jκ mice at approximately 3 months of age. Scale bars = 50 μm.

In order to assess the importance of RBP-Jκ in the endocrine differentiation program and the function of mature islets, RBP-Jκ was deleted from the endocrine progenitors (*Ngn3-Cre; RBP-Jκ*^*f*/*f*^*; YFP*). The Yellow Fluorescent Protein (YFP) reporter^33^ provides a stronger fluorescent signal than the EGFP from the *Z*/*EG* mice, and can be used in a similar manner to follow the fate of the Cre-expressing progenitors. In control mice, RBP-Jκ was expressed in both adult acinar and islets cells (Fig. 2a). While RBP-Jκ is usually described as being part of transcriptional activator/repressor complexes, its activity can be modulated through cytoplasmic export^34^. In control mice, RBP-Jκ was detected in the cytoplasm of most islet cells and in the nucleus of a small fraction of them (Fig. 2a). As expected, RBP-Jκ expression was absent in islets from *Ngn3-Cre; RBP-Jκ*^*f*/*f*^*; YFP* mice, (Fig. 2a). Similar to *Ngn3-Cre; Ps1*^*f*/*f*^*; Ps2*^−/−^*; RBP-Jκ/*^*f*^*; Z*/*EG* mice, the fluorescent reporter was expressed in the adult islets indicating that despite preventing all Notch signaling, *RBP-Jκ* deletion does not redirect Ngn3 progenitors towards the acinar fate as opposed to the Presenilin mice (Fig. 2a). Assessment of fasting glucose levels showed that *Ngn3-Cre; RBP-Jκ*^*f*/*f*^*; mice* exhibited glucose levels that were comparable to those of controls. (Fig. 2b). Moreover, in both males and females, glucose excursion and sensitivity in *RBP-Jκ*-deleted mice did not differ in any way from the control mice (male data in Fig. 2c,d, female data not shown). β-cells mass was also not affected (not shown).

### The pancreas from *Ngn3-Cre; RBP-Jκ* ^*f*/*f*^ mice exhibit areas of disorganized and undifferentiated cells

Unexpectedly, pancreas histology revealed that approximately half of the mice exhibited areas containing disorganized morphology with cells expressing the fluorescent reporters (5 out of 11 adult mice; Fig. 2e). The disorganized areas covered up to more than half of the pancreatic tissue in two of the mice, while the rest of the acinar and endocrine tissue was phenotypically normal. Importantly, deletion of *RBP-Jκ* did not redirect the endocrine progenitors away from the endocrine fate and towards the acinar lineage as observed in the Presenilin model (Fig. 2e). In adult mice, proliferation (Ki67) and apoptosis (Terminal Uridine Nick-End Labeling (TUNEL)) in the disorganized areas did not differ from the rest of the pancreas (not shown). The total pancreatic weight was also not affected in these mice (Fig. 2f).

### Embryonic origin of the disorganized masses found in the adult

**Figure 3:**
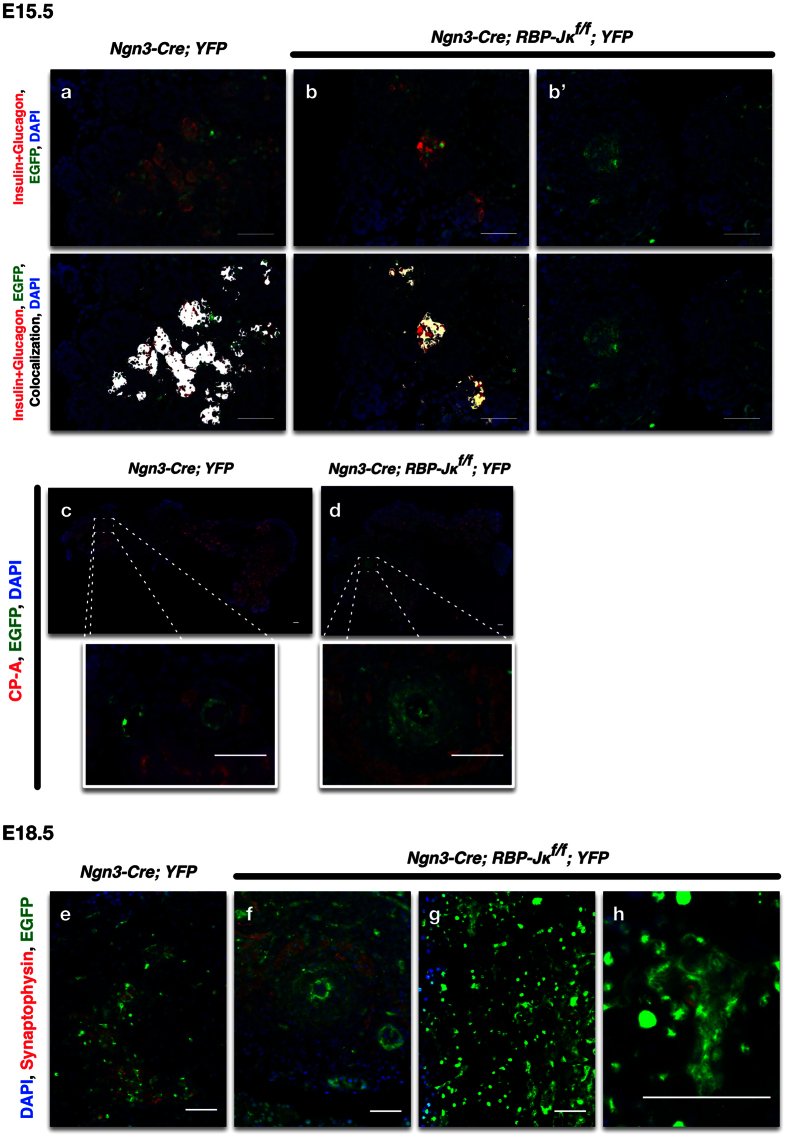
Lineage tracing of RBP-Jκ deficient Ngn3 progenitors during development. (**a**-**b’**) Combined immunostaining for Insulin and Glucagon (red), YFP (green), on controls (**a**) and RBP-Jκ (**b**-**b’**) pancreatic anlages at E15.5. The lower panels presents the same images with an overlay for the colocalization of Insulin + Glucagon in white. (**d**) Immunostaining for CP-A (red) and YFP (green) on E15.5 pancreatic rudiments of control (**c**) and RBP-Jκ mice with close-ups of the highlighted areas underneath (**d**). Immunostaining on E18.5 pancreatic sections for Synaptophysin (red) and YFP (green) in control (**e**) and RBP-Jκ mice (**f**–**h**). All nuclei were counterstained with DAPI (blue). Scale bars = 50 μm.

We then investigated the cell origin of these undifferentiated areas. These cells could originate from cells that expressed Ngn3, but because they did not have RBP-Jκ, could not differentiate properly to give rise to endocrine cells and remained in that stage. They could also be cells that differentiated into mature cells, but (because they did not have RBP-Jκ) eventually dedifferentiated to give rise to these disorganized masses. In order to identify the origin of these cells, we first assessed the appearance of these cells at different stages of development. At E13.5, only a few rare cells express the reporter and the control and RBP-Jκ mice are indistinguishable (not shown). At E15.5, when Ngn3 expression peaks, a number of cells that express the reporter can be detected in the developing pancreas (Fig. 3). In both control and RBP-Jκ mice, a number of progenitor cells already express either Insulin or Glucagon, though a number of scattered YFP+ cells that do not yet express either marker can also be observed in the RBP-Jκ mice (Fig. 3a,b’), often grouped in circular masses (Fig. 3b’). None of the YFP+ cells ever express acinar markers (Fig. 3c,d, with a close-up underneath). At the end of the secondary transition, at E18.5, when the differentiation of the endocrine progenitors peaks, most of the cells that express the reporter are now expressing the endocrine marker Synaptophysin (Fig. 3e–h) and are clustered into endocrine structures (Fig. 3e,f) in control mice. In the RBP-Jκ mice, while endocrine cells express the reporter, a number of undifferentiated YFP+ cells can still be observed grouped outside the forming islets (Fig. 3g,h).

### Sox9 expression is maintained in some of the RBP-Jκ deficient cells

**Figure 4:**
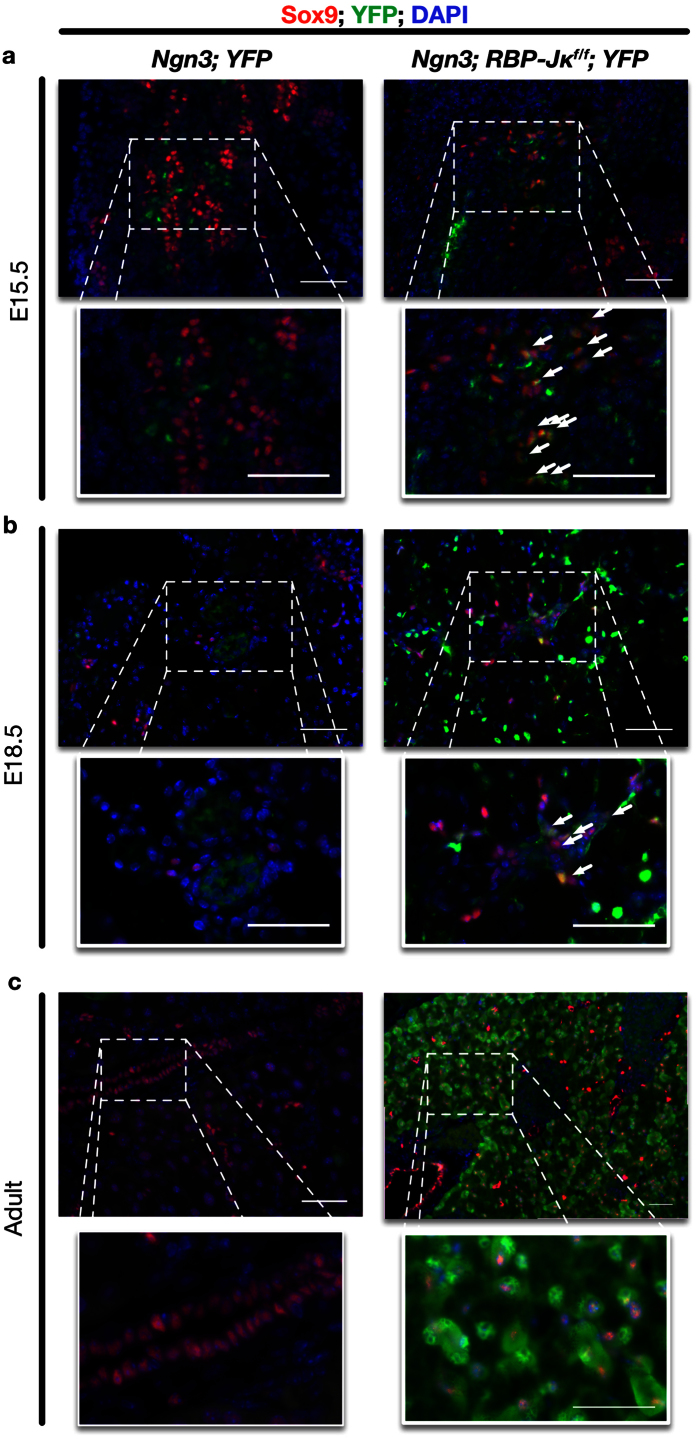
Characterization of the undifferentiated progenitors during development and in the adult. (**a**) Immunostaining for Sox9 (red), YFP (green) on control (left) and RBP-Jκ (right) E15.5 pancreatic rudiments. The lower line presents enlargements of selected areas. (**b**) Immunostaining for Sox9 (red), YFP (green) on *Ngn3-Cre; YFP* (left) and *Ngn3-Cre; RBP-Jκ*^*f*/*f*^*; YFP* (right) pancreatic anlages at E18.5. The lower line presents enlargements of selected areas. (**c**) Immunostaining for Sox9 (red), YFP (green) on disorganized areas of adult *Ngn3-Cre; RBP-Jκ*^*f*/*f*^*; YFP* mice (left). The right panel presents enlargements of selected areas. Arrows illustrate YFP+ cells expressing high levels of Sox9. All nuclei were counterstained in blue with DAPI. Scale bars = 50 μm.

Sox9 is a direct Notch target^35^,^36^ expressed in early, undetermined pancreatic progenitors. While Sox9 is a key regulator of cell fate during early pancreatogenesis, its expression quickly decreases in Ngn3 cells reviewed in^37^. At E15.5 and E18.5, the protein cannot be detected in the cells derived from the endocrine progenitors (controls in Fig. 4a,b). In *Ngn3-Cre; RBP-Jκ*^*f*/*f*^*; YFP* mice, while a number of YFP-positive cells express neither acinar nor endocrine markers, Sox9 is maintained at higher levels (Fig. 4a,b, arrows). Sox9 is also still highly expressed in the disorganized masses in the adult *Ngn3-Cre; RBP-Jκ*^*f*/*f*^*; YFP* mice (Fig. 4c).

### The disorganized areas in *Ngn3-Cre; RBP-Jκ* ^*f*/*f*^ mice revealed chronic pancreatitis, and acinar to ductal metaplasia

**Figure 5:**
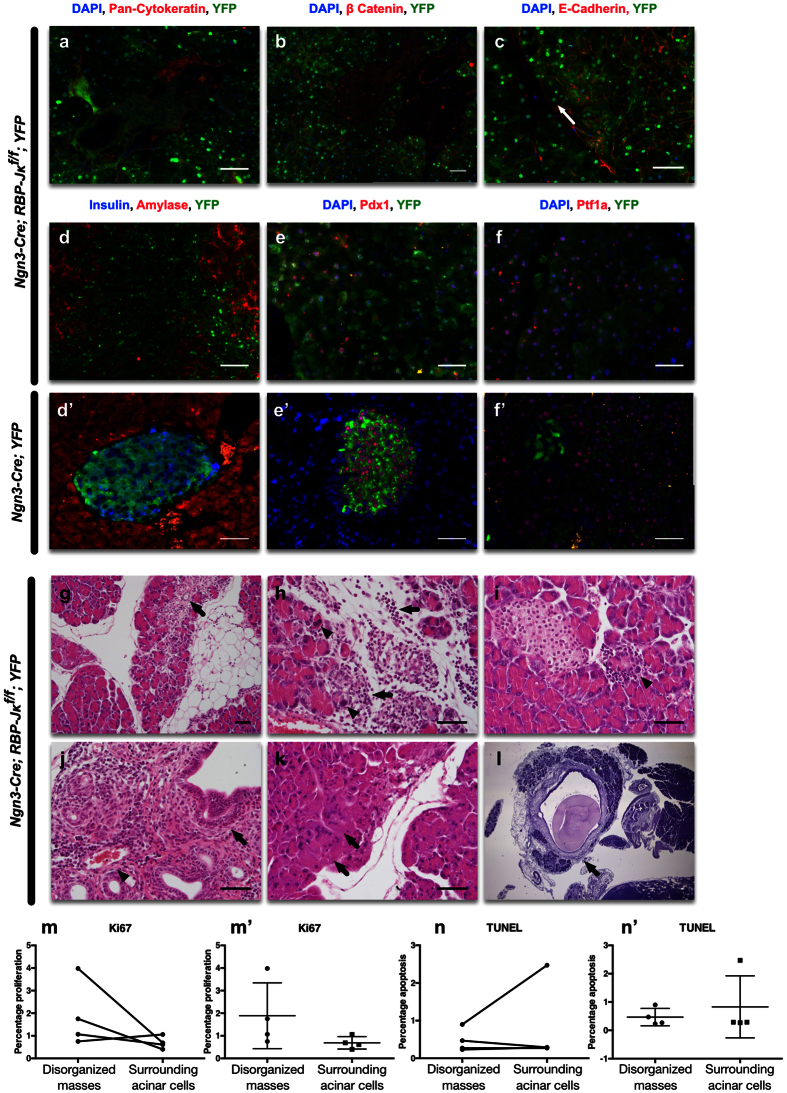
Histological assessment of the disorganized structures in the RBP-Jκ mice. Immunostaining on RBP-Jκ mice with YFP (green), and either (**a**) pan-Cytokeratin (red), (**b**) β-Catenin (red), (**c**) E-Cadherin (red). (**d**) Insulin (blue) and Amylase (red); (**e**) Pdx1 (red); or (**f**) Ptf1a (red). All nuclei were counterstained in blue with DAPI. (**g**–**l**) Hematoxylin and eosin staining on the adult *Ngn3-Cre; RBP-Jκ*^*f*/*f*^ *Z*/*EG* mice that present disorganized masses. H&E staining showed: (**g**) focal or spotty injury/necrosis of acinar cell and acinar to ductal metaplasia (arrow) in acinar parenchyma, accompanied by focal fibrosis and inflammatory infiltrates in the stroma; (**h**) acinar cell injury/necrosis (arrow heads) and inflammatory infiltrates (arrows) shown at high magnification; (**i**) focal acinar injury/necrosis surrounded by inflammatory response (arrow). (**j**) Periductal (main and interlobular) (arrow) and perivascular (arrow head) inflammatory infiltrates, composed mainly of lymphocytes and a number of eosinophils; (**k**) degenerative acinar cells displayed multinucleated syncytia and abundant cytoplasm (arrows); (**l**) stromal fibrosis and dilated interlobular ducts containing protein plug (arrow) indicating chronic pancreatitis. (**m**) Assessment of the percentage of proliferating Ki67+ cells in the disorganized masses and in the adjacent acinar cells (represented unpaired in **m’**). (**n**) Assessment of the percentage of apoptotic TUNEL+ cells in the disorganized masses and in the adjacent acinar cells (represented unpaired in **n’**). Scale = 50 μm.

The histology of the disorganized areas in *Ngn3-Cre; RBP-Jκ*^*f*/*f*^*; YFP* mice did not resemble either islet, acinar or ductal structures. These cells did not express any endocrine markers (Insulin, Glucagon, Synaptophysin), or acinar markers (Amylase or Carboxypeptidase-A (CP-A)). They were also negative for Lectin *Dolichos biflorus* agglutinin (DBA), Ngn3, Pax6, or MafA (Insulin and Amylase illustrated in Fig. 5d; other staining not shown). However, they expressed high levels of pan-Cytokeratin, E-Cadherin and β-Catenin, confirming the epithelial origin of these cells (Fig. 5a–c) and were positive for Pdx1 (Fig. 5e) and Ptf1a (Fig. 5f). E-Cadherin, outlines the borders of the cells, illustrating the different sizes and shapes within these structures with some of the cells forming multinucleated structures (white arrow in Fig. 5c).

In order to assess the progression of these structures in the pancreas over time, we studied 12 month-old mice. At this stage, the animals showed no difference in weight or glucose tolerance, suggesting that these abnormalities had no effect on glucose homeostasis and overall weight gain. Histological analysis in hematoxylin and eosin stained sections demonstrated that disorganized structures exhibited signs of chronic pancreatitis with focal or spotty acinar cells injury (arrowheads in Fig. 5g–i), necrosis, acinar to ductal metaplasia (ADM, arrows in Fig. 5g), surrounded by fibro-inflammatory stroma composed mainly of lymphocytes, infiltrating into injured parenchyma, adipose tissue (arrows in Fig. 5h). Periductal (main and interlobular; arrow) and perivascular (arrowhead) inflammatory infiltrates could also be seen with a number of eosinophils infiltrates throughout the tissue in Fig. 5j, indicating sub-acute inflammation. Some occasional multinucleated syncytia with a low nuclear-cytoplasmic ratio could also be observed (see arrows in Fig. 5k). Two of the older mice (one year-old) also presented pseudocyst, stromal fibrosis and dilated interlobular ducts containing protein plugs (arrow in Fig. 5l) indicating chronic pancreatitis. Proliferation and apoptosis were also assessed in the disorganized masses. While this data is only reflective of the stage at which the animal has been analyzed, it can reflect on the dynamic of the cells in the disorganized masses. In order to avoid any bias related to the age, gender or nutritional status of the mice, in each case, the proliferation or apoptosis percentage was compared to adjacent acinar tissue. Out of four animals, only one displayed a higher proliferation rate (Fig. 5m,m’ and illustrated in Suppl. Fig. 1). The percentage of TUNEL positive cells remained low in all mice (Fig. 5n,n’).

### The disorganized masses are not derived from the acinar lineage

**Figure 6:**
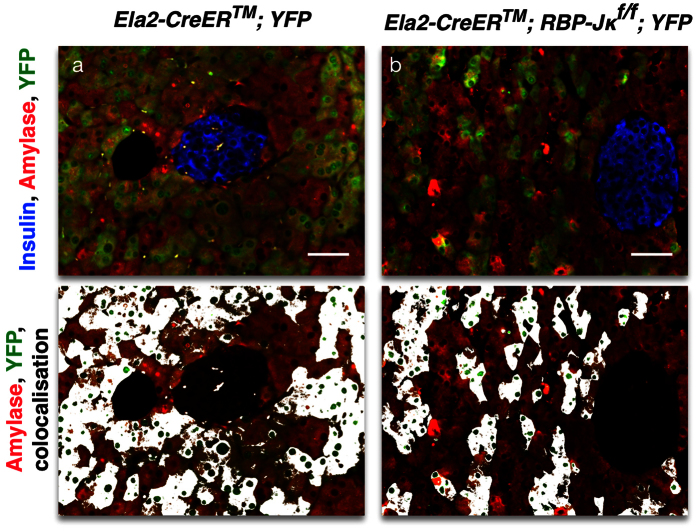
Lineage-tracing of RBP-Jκ deficient mature acinar cells. Confocal imaging of immunostaining for Insulin (blue), Amylase (red) and YFP (green) on control (**a**) and *Ela2-CreER*^*TM*^*; RBP-Jκ*^*f*/*f*^*; YFP* (in **b**) mice. The lower panels present the Amylase and YFP staining and overlays the colocalization in white. Scale = 50 μm.

Since Notch signaling is also required for exocrine regeneration^38^, it remained a possibility that some of the cells that were not expressing acinar or endocrine markers still at E18.5 could eventually give rise to acinar cells, and ultimately dedifferentiated into the disorganized masses observed in the adult. In order to test this hypothesis, we generated mice with a conditional, elimination of RBP-Jκ in the mature acinar cells using a Tamoxifen-inducible Cre driven by the Elastase 2 promoter^39^ (Ela2). Mice were administered Tamoxifen at 3 months of age and followed for 12 weeks before analysis. As illustrated in Fig. 6a, in control *Ela2-CreER*^*TM*^; *YFP* mice, the reporter is exclusively found in the acinar tissue. Similarly, in *Ela2-CreER*^*TM*^*; RBP-Jκ*^*f*/*f*^*; YFP* mice, the reporter was also restricted to the acinar compartment, and no disorganized or dedifferentiated masses are ever found (Fig. 6b). These results indicated that the disorganized masses found in the adult *Ngn3-Cre; RBP-Jκ*^*f*/*f*^*; YFP* mice were unlikely to come from dedifferentiated acinar cells.

### RBP-Jκ still controls the terminal endocrine fate in *RIP-Cre; RBP-Jκ* ^*f*/*f*^ mice

Recent publications demonstrated the plasticity of the mature endocrine cells^40^,^41^,^42^. Notch signaling is involved in the control of maturity in different models, including the acinar cells^38^,^43^,^44^,^45^. It remains a possibility that in the absence of RBP-Jκ (and therefore Notch signaling) in the Ngn3-Cre mice, some of the mature endocrine cells could dedifferentiate, lose all endocrine characteristics and form the disorganized masses observed in the *Ngn3-Cre; RBP-Jκ*^*f*/*f*^*; YFP* mice. In addition, the undifferentiated masses express Pdx1, which, in adult animals, is typically only observed in the β-cells. In order to test this hypothesis, mice with a deletion of RBP-Jκ in the β-cells were generated (*RIP-Cre; RBP-Jκ*^*f*/*f*^*; YFP*). In these mice, RBP-Jκ is deleted in nascent β-cells with the activation of the Insulin promoter.

**Figure 7:**
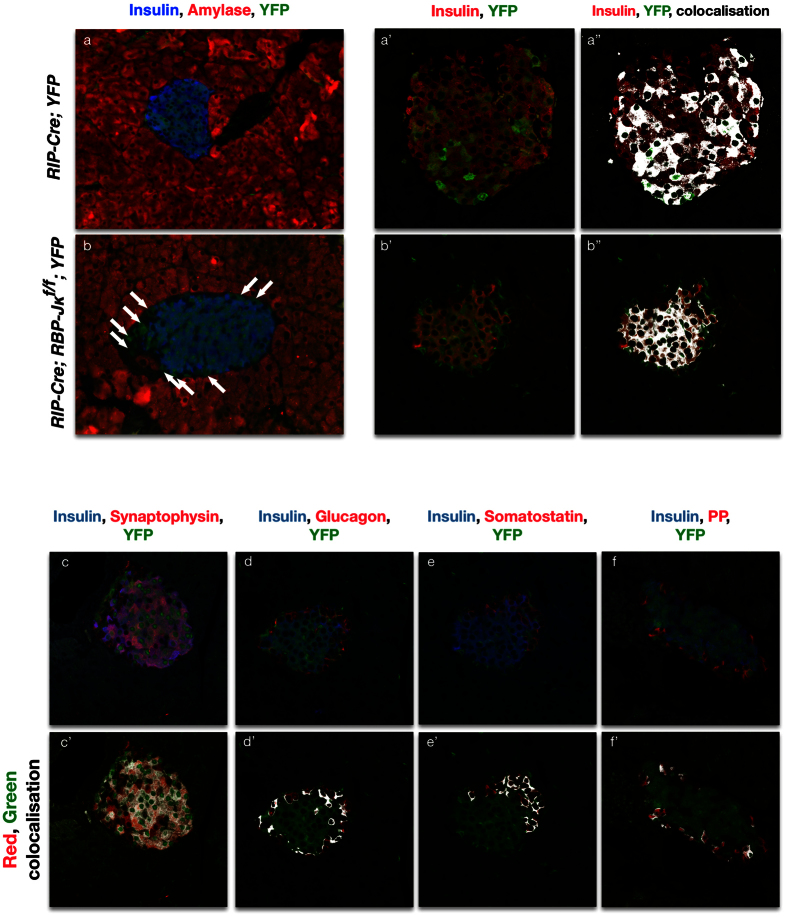
Lineage-tracing of RBP-Jκ deficient β-cells. Immunostaining for Insulin (blue), Amylase (red) and YFP (green) on *RIP-Cre; YFP* control (**a**) and *RIP-Cre; RBP-Jκ*^*f*/*f*^*; YFP* (in **b**) mice. Panels **a’**,**b’** present confocal imaging for Insulin (red) and YFP (green). Panels **a”**,**b”** display an overlay of the colocalization of the two channels in white. Panels (**c**–**f**) show confocal images for immunostaining for Insulin in blue and YFP in green with either Synaptophysin (**c**), Glucagon (**d**), Somatostatin (**e**) or Pancreatic Polypeptide (**f**) in red, with the corresponding overlay representation for the red and green channels in white in the corresponding panels below (**c’**–**f’**). Scale bars = 50 μm.

In the adults, the YFP reporter can be detected in all β-cells (Fig. 7a,b). Surprisingly though, a number of YFP+ positive cells do not express Insulin. Confocal microscopy and colocalization analysis illustrated that these cells still express Synaptophysin, and were therefore still within the endocrine lineage (Fig. 7c,c’). The Insulin-negative, YFP positive cells now expressed either Glucagon, Somatostatin or Pancreatic Polypeptide (Fig. 7d–f). This suggests that RBP-Jκ is contributing to the maintenance of the fate not only in pluripotent early endocrine progenitors, but also at least in a subset of the early β-cells. No disorganized masses or signs of dedifferentiation were found in these mice.

### β-cell dedifferentiation does not play a role into the formation of disorganized structures in *Ngn3-Cre; RBP-Jκ* ^*f*/*f*^ mice

In order to further test the timing during which RBP-Jκ could still help maintain the proper β-cell lineage, mice with a Tamoxifen-inducible Rat Insulin Promoter-Cre (*RIP-CreER*^*TM*^) were used to conditionally delete RBP-Jκ in mature β-cells (*RIP-CreER*^*TM*^*; RBP-Jκ*^*f*/*f*^*; YFP*). The deletion of RBP-Jκ was triggered by Tamoxifen injection at 3 months of age. After 12 weeks, the distribution of the reporter was once again analyzed to account for the maintenance of the fate of the deleted cells.

As expected, in the control *RIP-CreER*^*TM*^*; YFP* mice, the reporter was found exclusively in the β-cells (Suppl. Fig. 2a). A similar phenotype could be observed in the *RIP-Cre; RBP-Jκ*^*f*/*f*^*; YFP* mice (Suppl. Fig. 2b). As in the control mice, the expression of the reporter was restricted to cells co-expressing insulin. The reporter was never observed in other endocrine cell types and no disorganized masses were ever observed either. Islets always displayed a homogeneous Insulin staining implying the absence of dedifferentiation. Taken together, these data indicate that the disorganized masses do not originate from dedifferentiated β-cells from either developing or adult RBP-Jκ mice.

## Discussion

The present study was designed to evaluate how Notch signaling controls the maintenance of the endocrine progenitors fate and highlights the central role of Notch signaling, through RBP-Jκ, in controlling proper endocrine differentiation. A previous publication had illustrated that Notch signaling, through the Presenilins, was required for the maintenance of the fate of the endocrine progenitors in their original endocrine lineage^7^. In the absence of the Presenilins, Ngn3 progenitors were redirected towards the acinar lineage. The present study illustrates that RBP-Jκ is central to this process. In its absence, Presenilin-deficient Ngn3 progenitors can again properly form endocrine islets. In addition, we demonstrated that RBP-Jκ deletion in endocrine cells alters pancreas architecture while dispensable for glucose homeostasis. Unsuspectedly, RBP-Jκ deletion in Ngn3 endocrine progenitors also resulted in focal areas of undifferentiated cells originating during pancreatic organogenesis, with evidence of chronic pancreatitis and metaplasia in the adult, illustrating that RBP-Jκ was also involved in the proper maturation and differentiation of the endocrine progenitors. These phenotypes bear significant resemblance to those observed in models of HNF6 or Jagged1 deletions^46^,^47^,^48^ or in mice with a conditional elimination of RBP-Jκ using a Pdx1-Cre^49^.

In all three models, undifferentiated disorganized masses can be observed, though the deletions leading to these phenotypes took place much earlier during pancreatogenesis earlier during pancreatogenesis, and before Ngn3 itself was even expressed.

**Figure 8:**
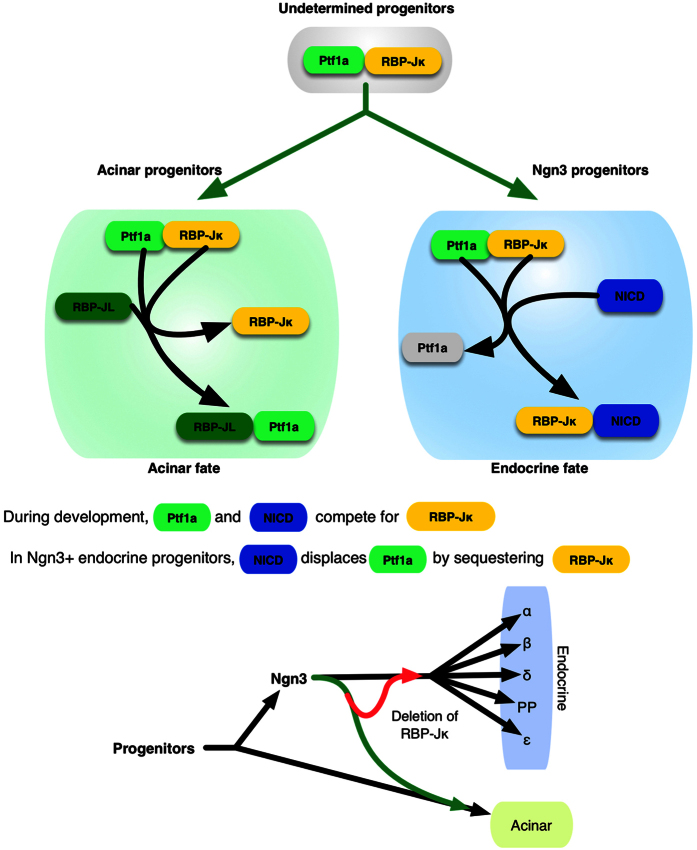
Diagram of the control of the endocrine fate by RBP-Jκ. During development, deleting RBP-Jκ from Ngn3 progenitors blocks Notch signaling but doesn’t push the cells towards the acinar fate since the Ptf1a/RBP-Jκ complex cannot form and promote the acinar differentiation pathways. Some of the Notch-deleted, Ngn3 progenitors fail to differentiate express high levels of Sox9 and form disorganized masses.

While Notch activation is prevented with the conditional elimination of the Presenilins in the Ngn3 progenitors, the additional removal of RBP-Jκ itself allows the restoration of the endocrine fate in these progenitors. Lineage tracing reveals that the Ngn3 progenitors can again form normal islets (Fig. 1). The mosaicism of the Cre has to be taken into account here. Ngn3 is expressed only during a very short amount of time and as the number of target alleles increases, so does the risk that some alleles will be missed. This is illustrated in Fig. 1 where 7 alleles need to be deleted for instance: while the deletion of RBP-Jκ allows progenitors to be maintained in their normal endocrine lineage again, green cells can be observed in the acinar compartment. Ptf1a is still expressed in Ngn3 progenitors^7^ and therefore we can hypothesize that when Notch is not actively sequestering RBP-Jκ, it can bind to Ptf1a and promote the acinar lineage through the activation of RBP-JL and the Ptf1a/RBP-JL complex^50^,^51^,^52^,^53^ (see diagram Fig. 8). This possibility is further reinforced by the fact that sole removal of RBP-Jκ from Ngn3 cells, while blocking Notch activity, also prevents the redirection of the fate of the endocrine progenitors (Fig. 2). With all Cre lines, in addition to mosaicism, the reliability of the Cre, remains an important question. We have consistently used Ngn3-Cre mice with a reporter for lineage tracing as a control for all our experiments and have never observed any misexpression of the reporter in the pancreas. While the BAC-Cre Ngn3 transgenic line has been reported to be expressed in the spermatogonia^54^, we have never observed generalized expression of the reporter in any of our mice, even when the father was carrying both the Cre and the reporter transgene. That being said, it remains a theoretical possibility that the Cre could have been expressed outside of its normal endocrine lineage. If that were the case, the main conclusions of the findings presented here would not be affected, as the conditional deletion of RBP-Jκ from the Ngn3 progenitors, while preventing Notch activation, no longer forces the progenitors towards the acinar fate.

RBP-Jκ-deleted Ngn3 progenitors form disorganized and undifferentiated masses in the adult pancreas and eventually lead to inflammation and acinoductal metaplasia. Lineage tracing demonstrated that these cells originate during development where undifferentiated masses still expressing high levels of Sox9 can be observed. Further experiments excluded the possibility that they could originate from either mature acinar (Fig. 6) or β-cells (Fig. 7 and Suppl. Fig. 2). It therefore appears that some level of RBP-Jκ is necessary for proper maturation of the early progenitors. While Sox9 levels (in the ducts for instance) have previously been associated with active Notch signaling^55^, in different Systems, Notch, through RBP-Jκ, is a known potent direct inhibitor of Sox9^35^,^36^. In the absence of Notch signaling, Sox9 levels remain high in the Ngn3 progenitors (Fig. 4) and could delay, or prevent proper maturation. Additional Notch targets could also contribute to this mechanism, and the deletion of RBP-Jκ in itself could lead to the derepression of some of these targets. Considering the half-life of RBP-Jκ and the precise timing at which the floxed alleles are deleted, some level of proteins could still be present shortly after Ngn3 is expressed: most cells could then form proper islets while cells with lower levels of RBP-Jκ would remain undifferentiated. The deletion of RBP-Jκ also prevents the formation of the Ptf1a/RBP-Jκ complex, which could explain the progression of acinar to ductal metaplasia as recently described with deletions of Ptf1a^56^. The acinoductal metaplasia and cell fusions would lead to suspect that the animals could be prone to developing tumors. Considering the severity of the transformations, the absence of RBP-Jκ could be seen as a “first hit” that could eventually lead to malignant transformations, though no tumors have yet been observed in these animals (for up to 16 months). The different genetic models presented here suggest that the control of the fate of endocrine progenitors by RBP-Jκ is progressively lost as they mature. While Ngn3 progenitors without RBP-Jκ could completely fail to differentiate (Figs 2, 3, 4, 5), the control over the fate of the cells seems restricted to the endocrine lineage when RBP-Jκ is deleted after Insulin is expressed (Fig. 7), while the RBP-Jκ protein seems to be completely dispensable in mature β-cells (Suppl. Fig. 2).

One important finding was that elimination of RBP-Jκ from the endocrine progenitors during development had no effect on glucose homeostasis in the adult. A previous study had hinted that RBP-Jκ might not be required in mature β–cells^57^, but through lineage-tracing, we were able to demonstrate that Ngn3 progenitors without RBP-Jκ can form functional islets. This interesting finding suggests that RBP-Jκ-dependent transcription is less important for mature islet cells and that the transcriptional activity of RBP-Jκ is limited to embryonic stages. The islets are undistinguishable from the control mice (see Fig. 2a), which is further confirmed by eliminating RBP-Jκ from mature β-cells, as illustrated in Fig. 6 and previously indicated without lineage-tracing by Fujikura *et al*.^26^. These mice are also perfectly glucose responsive (see Fig. 2b,c). While the animals have not been placed under higher metabolic stress (high fat feeding, streptozotocin), this would indicate that even though RBP-Jκ is central to the maintenance of the fate of the Ngn3 progenitors right after Ngn3 has been expressed, it might be dispensensable under normal physiological conditions.

The previous view on the control of the fate of the pancreatic Ngn3 progenitors placed the Presenilins at the center of the maintenance of the endocrine fate. Collectively, our data now highlight how Notch activation is required to take away RBP-Jκ and prevent the reactivation of the acinar program. RBP-Jκ needs to be sequestered away from the acinar-promoting transcription complex to allow for the maturation of islets. In addition, RBP-Jκ also seems to be playing a central part in the progression of the proper differentiation of the islet progenitors, and its deletion can altogether maintain them in an undifferentiated state up until adulthood.

## Methods

### Animal generation

The *Ngn3-Cre*^58^ and *RIP-CreER*^*TM*^^59^ mice were obtained through a generous gift from D. Melton. The *Ps1*^*f*/*f*^^60^, *Ps2*^−/−^61, *RBP-Jκ*^*f*/*f*^^62^ and *Ela2-CreER*^*TM*^ mice were obtained through generous gifts of J. Shen, B. DeStrooper, T. Honjo and J. Williams respectively. The *Z*/*EG* and *CAG-YFP* (abbreviated as YFP) reporter mice were obtained from The Jackson laboratory (Bar Harbor, ME). Depending on the embryonic stages, fixation and staining conditions, the EGFP reporter expressed in the *Z*/*EG* mice is sometimes weakened and difficult to observe so the *YFP* reporter mice were used instead^63^. The respective *Cre* mice were used as controls for all the experiments. Mice were maintained on a mixed C57Bl6 background and interbred to obtain the necessary combined genotypes. Genotyping of all mice was performed by PCR. All experiments with mice were conducted in accordance procedures approved by the University of Michigan Animal Studies Committees. Unless explicitly specified, all experiments on adult mice were conducted at approximately 3 months of age (between 11 and 14 weeks).

### Tamoxifen administration

Tamoxifen (Sigma, St Louis, MO) was resuspended in corn oil and administered through 5 intra-peritoneal daily injections at 5 mg/kg as previously described^40^.

### Tissue preparation and Immunostaining

Tissues were fixed in 3.7% formalin in PBS for durations ranging from 20 minutes to overnight depending on the stage and size of the tissue. Smaller tissues were embedded in Histogel (Thermo Scientific, Kalamazoo, MI) before paraffin processing and embedding. Formalin-fixed pancreatic tissues were embedded in paraffin using standard techniques. 5 μm sections were collected and analyzed for immunostaining or hematoxylin/eosin. Slides were subjected to antigen retrieval using citrate buffer and permeabilized with 0.1% triton.

The primary antibodies were used overnight at the given dilution (Suppl. Methods Table 1). Sections were incubated with the appropriate secondary antibodies conjugated to Fluorescein isothiocyanate (FITC), Aminomethylcoumarin (AMCA) or Cy3 (Jackson Immunoresearch, West Grove, PA). When necessary, nuclei were counterstained with DAPI (Vector Laboratories, Burlingame CA). TUNEL staining was performed following the manufacturer’s instructions (Abcam, Cambridge, MA).

Fluorescent images were acquired using a Leica microscope DM5500B with a motorized stage using a Leica DFC360FX camera (Leica Microsystems, Wetziar, Germany), interfaced with the OASIS-blue PCI controller and controlled by the Surveyor software (version 7.1, Objective Imaging Ltd, Cambridge, UK). Confocal imaging was performed on an Olympus FluoView 500 Laser Scanning Confocal Microscope LSCM using the Fluoview software (Olympus Scientific Solutions Americas Corp., Waltham, MA).

### Colocalization analysis

Colocalization analysis was performed using ImageJ 1.49d^64^, with Pierre Bourdoncle’s colocalization plugin (Institut Jacques Monod, Service Imagerie, Paris, France, http://rsb.info.nih.gov/ij/plugins/colocalization.html).

### Morphometric analysis and cell counts

The entire pancreatic buds were sectioned at 5 μm thickness. For the *in vitro* studies every other section of the bud was stained for Pdx-1 and Ki67 analysis. Alternate sections were stained for Ngn3 and Ki67 analysis. Different parameters (total number, Ki67, Pdx1 and Ngn3) were counted using ImageJ 1.49o^64^ and the Image-based Tool for Counting Nuclei (ITCN) plug-in version 1.6 from the Center for Bio-Image Informatics (http://www.bioimage.ucsb.edu/automatic-nuclei-counter-plug-in-for-imagej).

### Metabolic studies

Blood samples were collected from the tail vein after an overnight fasting. Glucose was measured on whole blood using Accu-Chek Aviva (American Diabetes Wholesale, Pompano beach, FL). Glucose tolerance tests were performed by intraperitoneally injection of glucose (2 mg/g) after an overnight fast as previously described^65^.

### Statistical analysis

All values are expressed as mean ± standard error. Statistical analyses were conducted using unpaired, non-parametric Mann-Whitney test for unpaired statistics or a Wilcoxon match-pairs signed rank test for paired experimental designs using Prism 6.0f (GraphPad Software, La Jolla, CA). Results were considered significant with a *P* value ≤ 0.05.

## Additional Information

**How to cite this article**: Cras-Méneur, C. *et al*. Early pancreatic islet fate and maturation is controlled through RBP-Jκ. *Sci. Rep*. **6**, 26874; doi: 10.1038/srep26874 (2016).

## Supplementary Material

Supplementary Information

Supplementary Method Table 1

Supplementary Figure Legends
