# Supplementary material for: Early pancreatic islet fate and maturation is controlled through RBP-Jκ
Source: Sci Rep. 2016 May 31;6:26874. doi: 10.1038/srep26874 (PMC4886527; doi:10.1038/srep26874)

Early pancreatic islet fate and maturation is controlled through RBP-Jκ

Corentin Cras-Méneur, Megan Conlon, Yaqing Zhang, Marina Pasca Di Magliano, Ernesto Bernal-Mizrachi

Supplemental Figure 1

DAPI, Ki67, YFP

Ngn3-Cre; RBP-Jκ$^{f/f}$; YFP

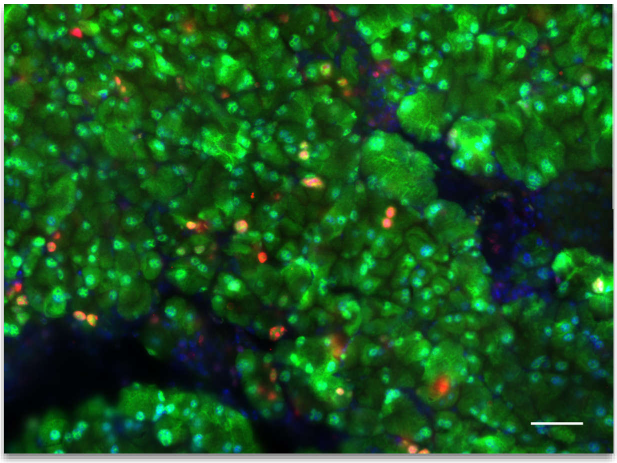

Early pancreatic islet fate and maturation is controlled through RBP-Jκ

Corentin Cras-Méneur, Megan Conlon, Yaqing Zhang, Marina Pasca Di Magliano, Ernesto Bernal-Mizrachi

Supplemental Figure 2

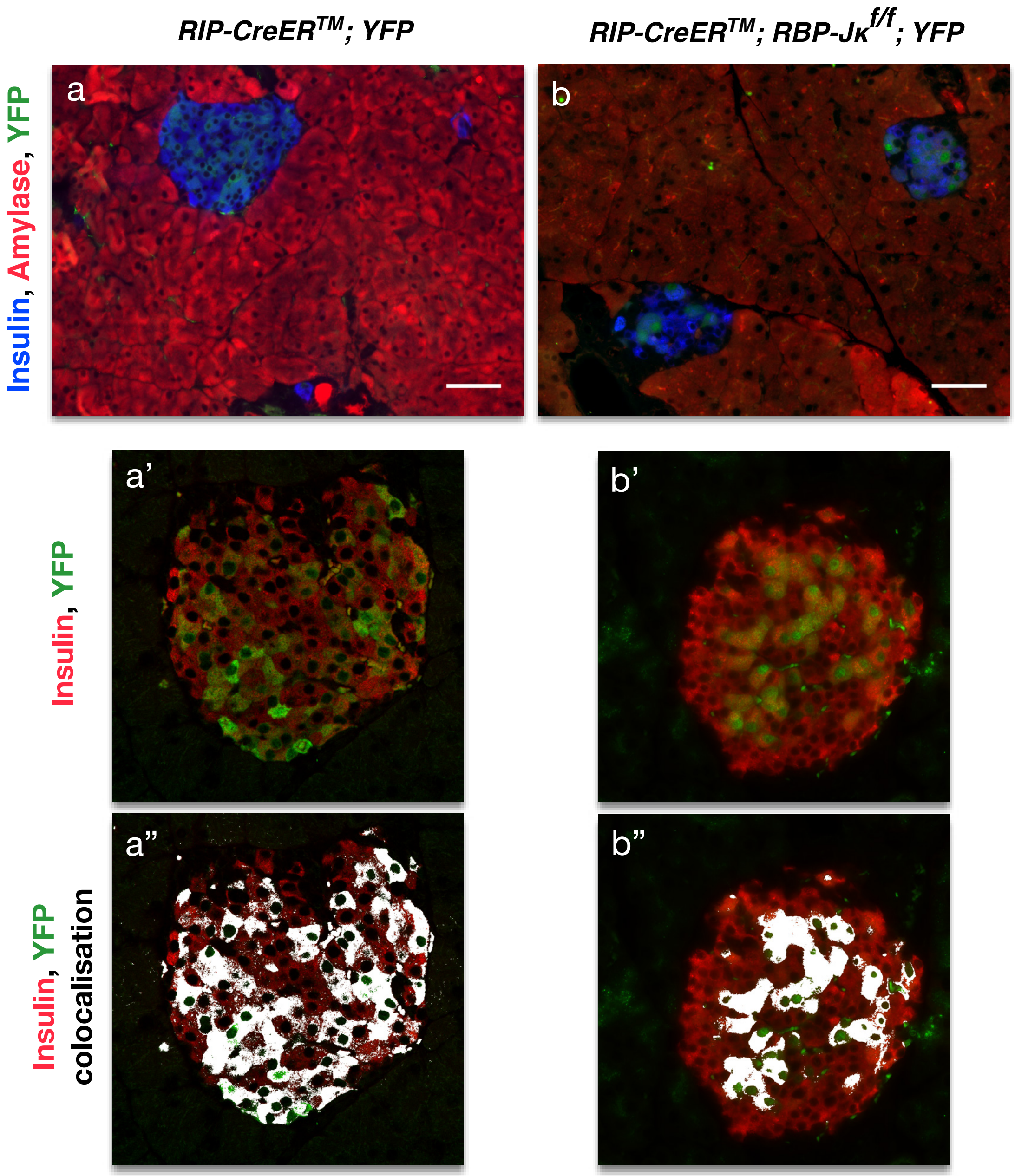

Supplement: Supplementary Information [file srep26874-s1.pdf]
