# Supplementary material for: Early pancreatic islet fate and maturation is controlled through RBP-Jκ
Source: Sci Rep. 2016 May 31;6:26874. doi: 10.1038/srep26874 (PMC4886527; doi:10.1038/srep26874)
Supplement: Supplementary Method Table 1 [file srep26874-s2.doc]

**Early pancreatic islet fate and maturation is controlled through RBP-Jκ**

Corentin Cras-Méneur, Megan Conlon, Yaqing Zhang, Marina Pasca Di Magliano, Ernesto Bernal-Mizrachi

##

## Supplemental Method Table 1: Antibody table.

| **Antibody** | **Company, City, State/Country** | **Dilution** |
| --- | --- | --- |
| Rabbit anti-Amylase | Sigma, St Louis, MO | 1:300 |
| Rabbit anti-Carboxypeptidase-A | Serotec, Kidlington UK | 1:300 |
| DBA (Dolichos Biflorus Agglutinin) | Vector Laboratories, Burlingame CA | Staining performed according to the manufacturer’s instructions |
| Rat anti-E-Cadherin | Invitrogen, Grand Island, NY | 1:200 |
| Mouse anti-Glucagon | Abcam, Cambridge, MA | 1:400 |
| Chicken anti-green fluorescent protein (EGFP and YFP) | Abcam, Cambridge, MA | 1:600 |
| Guinea-pig anti-Insulin | Dako, Glostrup, Denmark | 1:800 |
| Rabbit anti-Ki67 | Vector Laboratories, Burlingame CA | 1:200 |
| Rabbit anti-MafA | Bethyl Laboratories Inc., Montgomery, TX | 1:100 |
| Rabbit anti-Ngn3 | Chemicon, Temecula, CA | 1:200 |
| Mouse anti-pan-Cytokeratin | Sigma, St Louis, MO | 1:150 |
| Rabbit anti-Pax6 | Biolegend, Dedham, MA | 1:300 |
| Rabbit anti-Pdx1 | Millipore, Temecula, CA | 1:800 |
| Goat anti-Pdx1 | Santa Cruz, Dallas, TX | 1:100 |
| Rabbit anti-Ptf1a | Gift from B. Blondeau | 1:1000 |
| Rabbit anti-RBP-Jκ | Chemicon, Temecula, CA | 1:200 |
| Rabbit anti-Sox9 | Millipore, Temecula, CA | 1:600 |
| Rabbit anti-Synaptophysin | Abcam, Cambridge, MA | 1:200 |
| TUNEL Apoptosis Detection Kit | Millipore, Temecula, CA | Staining performed according to the manufacturer’s instructions |
| Rabbit anti-β-Catenin | Sigma, St Louis, MO | 1:1000 |
