# Supplementary material for: Early pancreatic islet fate and maturation is controlled through RBP-Jκ
Source: Sci Rep. 2016 May 31;6:26874. doi: 10.1038/srep26874 (PMC4886527; doi:10.1038/srep26874)
Supplement: Supplementary Figure Legends [file srep26874-s3.doc]

**Early pancreatic islet fate and maturation is controlled through RBP-Jκ**

Corentin Cras-Méneur, Megan Conlon, Yaqing Zhang, Marina Pasca Di Magliano, Ernesto Bernal-Mizrachi

##

## Supplemental Figure 1

Immunostaining for Ki67 (red) and YFP (green) with a DAPI counterstain (blue) on a selected area of a *Ngn3-Cre; RBP-Jκ^f/f^; YFP* mouse. Scale bars=50 μm.

## Supplemental Figure 2

Immunostaining for Insulin (blue), Amylase (red) and YFP (green) on control (**a**) and *RIP-CreER^TM^; RBP-Jκ^f/f^; YFP* (in **b**) mice. Panels **a'** and **b'** show confocal imaging for Insulin (red) and YFP (green). Panels **a"** and **b"** overlays the colocalization of the two channels in white. Scale bars=50 μm.
